# Adult ADHD patient experiences of impairment, service provision and clinical management in England: a qualitative study

**DOI:** 10.1186/1472-6963-13-184

**Published:** 2013-05-21

**Authors:** Lauren Matheson, Philip Asherson, Ian Chi Kei Wong, Paul Hodgkins, Juliana Setyawan, Rahul Sasane, Sarah Clifford

**Affiliations:** 1 Department of Clinical Health Care, Faculty of Health and Life Sciences, Oxford Brookes University, Oxford OX3 0FL, UK; 2 MRC Social Genetic and Developmental Psychiatry, Institute of Psychiatry, Kings College London, De Crespigny Park, Denmark Hill, London SE5 8AF, UK; 3 Centre for Safe Medication Practice and Research, Department of Pharmacology and Pharmacy, Li Ka Shing Faculty of Medicine, University of Hong Kong, Hong Kong, China; 4 Global Health Economics & Outcomes Research, Shire Development LLC., Wayne, PA 19087, USA; 5 Bayer Healthcare Pharmaceuticals, Pine Brook, NJ 07058, USA; 6 United BioSource Corporation, 7101 Wisconsin Avenue, Suite 600, Bethesda, MD 20814, USA

**Keywords:** Adult, ADHD, Qualitative, Experiences, Treatment, Impairment

## Abstract

**Background:**

There is limited evidence of the unmet needs and experiences of adults with Attention Deficit Hyperactivity Disorder (ADHD) in the published scientific literature. This study aimed to explore the experiences of adults in England with ADHD regarding access to diagnostic and treatment services, ADHD-related impairment and to compare experiences between patients diagnosed during adulthood and childhood.

**Methods:**

In this qualitative study, 30 adults with ADHD were recruited through an ADHD charity (n = 17) and two hospital outpatient clinics for adults with ADHD in England (n = 13). Half of the participants were diagnosed with ADHD during childhood or adolescence and the remainder during adulthood. Semi-structured interviews were conducted and data was analysed using a thematic approach based on Grounded Theory principles.

**Results:**

Analysis revealed five core themes: ‘An uphill struggle’: the challenge of accessing services, ‘Accumulated Psychosocial Burden and the Impact of ADHD’, ‘Weighing up Costs vs. Benefits of ADHD Pharmacological Treatment’, ‘Value of Non-pharmacological Treatment’ and ‘Barriers to Treatment Adherence’. Accessing services and the challenges associated with securing a definitive diagnosis of ADHD in adulthood was an ‘*uphill struggle*’, often due to sceptical and negative attitudes towards ADHD by healthcare professionals. ADHD-related impairment had an overwhelmingly chaotic impact on every aspect of patients’ lives and many felt ill equipped to cope. A persistent sense of failure and missed potential from living with the impact of ADHD impairment had led to an accumulated psychosocial burden, especially among those diagnosed from late adolescence onwards. In contrast, positive adjustment was facilitated by a younger age at diagnosis. Although medication was perceived as necessary in alleviating impairment, many felt strongly that by itself, it was inadequate. Additional support in the form of psychological therapies or psycho-education was strongly desired. However, few patients had access to non-pharmacological treatment. In some, medication use was often inadequately monitored with little or no follow-up by healthcare professionals, leading to poor adherence and a sense of abandonment from the healthcare system.

**Conclusion:**

The findings suggest that the unmet needs of adults with ADHD are substantial and that there is a wide gap between policy and current practice in England.

## Background

Attention deficit hyperactivity disorder (ADHD) is a neurodevelopmental disorder, which, until recent years, has been perceived as a disorder restricted to childhood [1]. Yet follow-up studies of children with ADHD clearly demonstrate that the disorder often persists into adulthood [2,3], affecting around 2-4% of adults in the UK and worldwide [1,4-7]. In the majority of patients diagnosed in childhood, ADHD impairment and symptoms continue to be problematic in adult life [3,8]. In adulthood, ADHD symptoms typically feature inattention, impulsivity, restlessness, disorganisation and forgetfulness, similar to characteristics in childhood [5,6,9]. This can result in significant impairments across multiple domains of adult life, affecting interpersonal relationships, family dynamics, education, occupation and overall health-related quality of life [1,10,11]. For instance, adults with ADHD are at increased risk of divorce or separation, unemployment, imprisonment and academic failure [10-12].

In addition, many adult patients with ADHD have gone unrecognised and undiagnosed in childhood and approach health services to seek help for the first time in adulthood [13]. ADHD is often misdiagnosed in these individuals, and the presence of co-existing disorders, such as depression or anxiety, may contribute further to this problem [5,13]. If ADHD is misdiagnosed, this can result in ineffective pharmacological and non-pharmacological treatments being prescribed, which in some cases may be detrimental to the individual and would not usually alleviate their ADHD-related symptoms. Furthermore, there is often a substantial psychosocial and functional burden associated with undiagnosed and untreated ADHD [14]. Yet there is very little understanding of the perspectives of adults with ADHD reported in the scientific literature [15,16], particularly regarding access to services and how experiences may differ between those diagnosed in childhood and those in adulthood. Previous studies in this patient population have mostly employed quantitative approaches [17,18] which are limited at investigating patients’ perspectives as they restrict the range of responses from participants. Qualitative approaches are more appropriate at providing an in-depth insight into patients’ experiences [19].

Recent guidelines by the National Institute of Health and Clinical Excellence (NICE) have recommended pathways of care for children and adults with ADHD in the UK [20,21]. In particular, NICE recommends the use of pharmacological treatments that have been shown to be effective in adults with ADHD, mainly the stimulants methylphenidate, dexamphetamine and atomoxetine [1,22]. In addition, NICE guidelines state that drug treatment should always be part of a comprehensive treatment program addressing psychological, behavioural and educational/employment needs [20,21]. However, no research to date has investigated patients’ experiences of accessing the range of ADHD treatments in England or patients’ attitudes and experiences regarding both pharmacological and non-pharmacological treatment. This is needed as it may suggest ways of promoting optimal functioning in adult patients with ADHD.

Therefore, this study aimed to better understand the health care and treatment-related experiences and needs of adults with ADHD, specifically, to explore: a) patients’ experiences of accessing services and receiving a diagnosis of ADHD; b) patients’ experiences of ADHD treatment, both pharmacological and non-pharmacological; c) patients’ experiences of ADHD-related impairment; and finally, d) to compare the experiences between adult patients diagnosed in childhood and in adulthood.

## Methods

### Methodological approach

A qualitative approach using semi-structured interviews was used as it enabled a detailed and in-depth exploration of patients’ experiences and views. Moreover, the technique is particularly useful at improving knowledge of poorly understood areas of health care [19,23]. Face-to-face interviews were chosen due to the sensitive and potentially distressing nature of the research topics.

### Sample

Participants were eligible for the study if they had received a diagnosis of ADHD (self-reported), were over 18 years old, living in England, and had previous or current experience of ADHD pharmacological treatment for a minimum duration of 3 months. Participants were ineligible if they were unable to participate in a face-to-face interview. Stratified sampling was used to recruit a total of 30 participants from two different subgroups of adult ADHD patients:

**Group 1**: Adult patients who were diagnosed and commenced pharmacological treatment for ADHD during childhood or adolescence (under age 18).

**Group 2:** Adult patients who were diagnosed and commenced pharmacological treatment for ADHD during adulthood (aged 18 or over).

Both groups included participants who had been on treatment without a break (continuous care), and others who had periods of at least 6 months without care or treatment (non-continuous care). In total, 15 participants were recruited where ADHD was first diagnosed in adulthood and 15 were recruited where the diagnosis was made during childhood or adolescence. This sample size is deemed sufficient in qualitative research [24] in terms of generating thematic saturation for most topics. Within each group, theoretical sampling was used to maximise the range of participants within each category, such as age, gender and symptom severity.

### Recruitment

**Figure 1:**
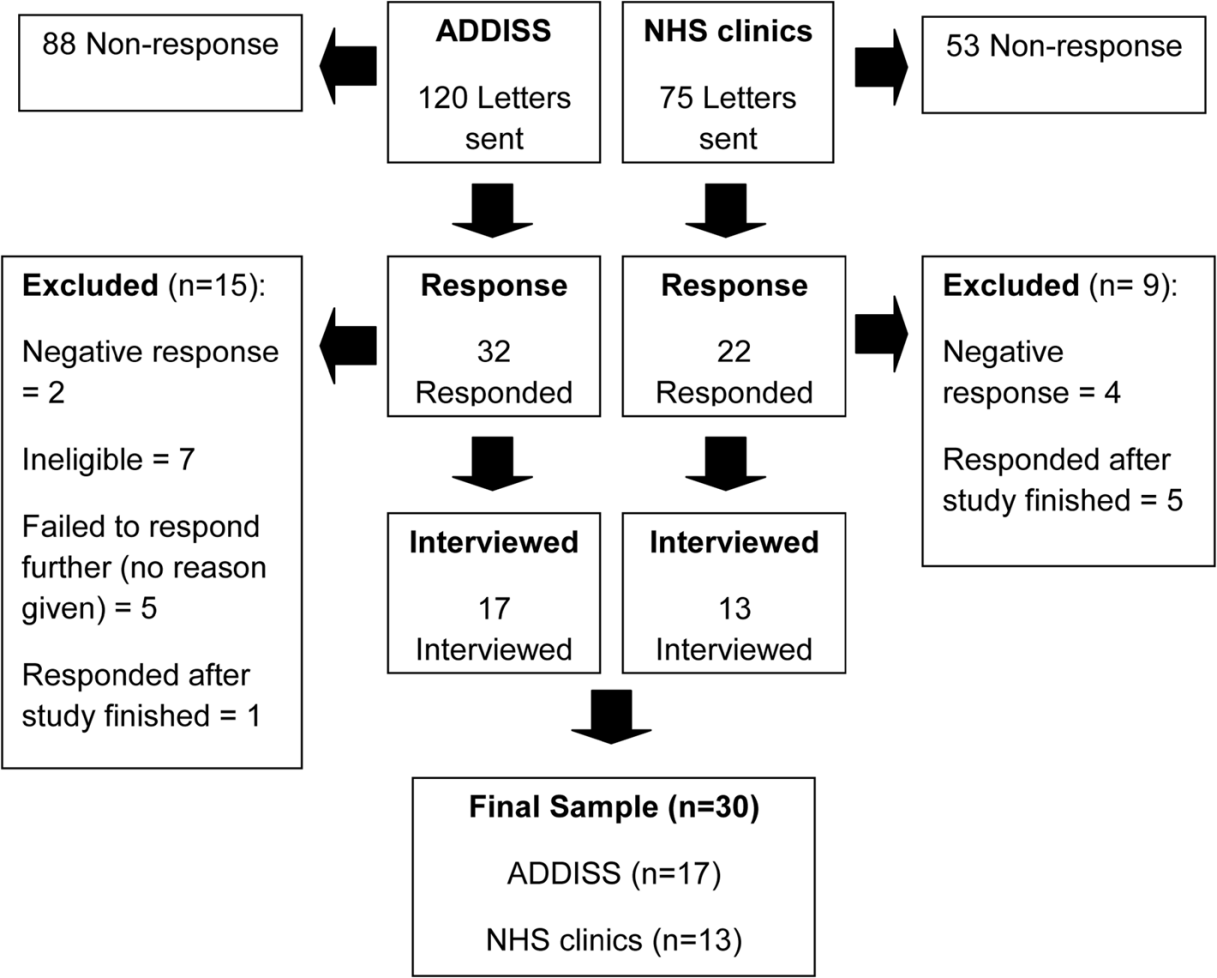
Study response rate.

Ethical approval was obtained from the Cambridgeshire 1 National Health Service (NHS) ethics committee (REC reference: 10/H0304/20**)** and research governance approval from appropriate NHS Trust sites. Patient interviews were conducted between December 2010 and June 2011. Participants were recruited via two methods and the process is summarised in Figure 1. Seventeen patients were recruited through the National Attention Deficit Disorder Information and Support Service (ADDISS) charity, which has nationwide members. Invitation letters were posted to 50 adults with ADHD and 70 parents of adults with ADHD, requesting them to pass on details of the study to their son or daughter. A freepost envelope was enclosed with the invitation letter. The study was also advertised in an online ADDISS newsletter and was also emailed out to the member database on two occasions. If participants indicated by post or email that they were willing to participate, a convenient date and location was arranged for the interview. Furthermore, 13 patients were recruited from two NHS outpatient clinics for adults with ADHD in London and the South East of England. The reason for recruiting from both a healthcare and non-healthcare setting was to try to capture a range of experiences and views and to minimise bias from recruiting from just one source.

### Data collection

Semi-structured face-to-face interviews were conducted in either the participant’s home (n = 15), in a private room at the School of Pharmacy in London (n = 12) or at an alternative convenient location (n = 3). A semi-structured interview guide was developed by a multi-disciplinary team including two psychologists, a pharmacist and a psychiatrist. This was used to guide the discussion in each individual interview, and enabled the interviewer to follow-up on any unanticipated issues that arose during interviews. Written informed consent was obtained from all participants prior to interview. Participants were reassured that the interviews were confidential and transcripts would be anonymised. The interview guide involved a series of open-ended questions, and topics included accessing services, treatment (non-pharmacological and pharmacological) and ADHD-related impairment. Prompts were used where necessary to elicit more detailed responses. Participants were free to respond in their own words and the researcher followed up on topics that arose outside of the interview guide. The interviews lasted approximately 1 hour and were digitally-recorded, with participants’ consent. Field notes were written up after each interview which recorded the researcher’s thoughts, feelings and reflections about the interview.

In order to describe the sample, participants also completed a brief survey to obtain self-reported demographic characteristics, prescription medication use, and ADHD symptoms and impairment. The symptom severity checklist is a validated questionnaire also known as the Adult ADHD Self Report Scale (ASRS v.1) [25]. The impairment checklist [26] is a non-validated questionnaire which asks participants to rate (scale of 1 to 5, with high scores indicating greater impairment) their level of impairment for different domains e.g. home life.

### Data analysis

#### Qualitative data

Interviews were transcribed verbatim and a general thematic approach was used to analyse the transcripts using key principles of Grounded Theory [27,28]. A Grounded Theory approach is an inductive methodology that allows theory to be developed from the systematic gathering and analysis of data. Each transcript was read and re-read several times, then initially coded line-by-line. This process was repeated for each transcript and then codes were defined and categorised into sub themes and meta themes. The analysis was an iterative process, as transcripts were constantly revisited when new themes emerged. Data collection and analysis occurred simultaneously, so that any initial themes or unanticipated topics arising from the data were followed up in subsequent interviews. Transcripts were then analysed as a whole by the process of constant comparison [28], and overarching themes emerged across the entire data set.

A reflexive research diary was kept during the entire research process by LM to record any thoughts regarding emerging codes or ideas resulting from the analysis [29]. To ensure reliability, two researchers (SC and LM) independently coded the first 4 interviews then 3 further randomly selected interviews as data collection progressed. After comparison and discussion of codes and understanding of the transcripts, there was a high degree of agreement on the major themes and sub themes. Some sub themes were phrased differently, but this was usually due to differences in terminology, rather than the meaning of emerging concepts. An expert psychiatrist (PA) specialising in ADHD in adults reviewed a summary of the initial themes and verified the themes for face validity. In order to further ensure validity, member checking [29] was conducted with a random sample of participants (n = 4), who were sent a short lay summary of their individual interviews. All respondents agreed that the interpretation of the interview was accurate. A qualitative software program (NVivo v.9) [30] was used to assist with organising the themes. A data saturation matrix was generated, which illustrated that thematic saturation was achieved as no new themes were emerging.

#### Quantitative data

Quantitative data was entered and analysed using SPSS v. 18 ™. As data were collected for descriptive purposes, only descriptive statistics were performed. Self-reported demographic characteristics and prescription medication use were reported as mean (SD) for continuous variables or n (%) for categorical variables. For the ASRS [25] scale, patients’ self-reported symptom ratings were recoded to 0 or 1, indicating symptoms consistent with ADHD according to the ASRS. Scores were also recoded to 0, 1, 2 or 3 to indicate ‘none’, ‘mild,’ ‘moderate’ or ‘severe’ ADHD symptoms. The Impairment scale was recoded to reflect the number of scores which indicated that ADHD impairment was definitely present for each of the 10 domains. The mean (SD) and range of the total scores are reported.

## Results

Thirty participants were included in this study. The sample included 15 participants diagnosed in childhood or adolescence (of which 8 had continuous care) and 15 participants diagnosed in adulthood (of which 8 had continuous care). There were 13 males and 17 females with an age range of 18 to 57 years (mean age 34.9 years, SD = 14.6). The response rate from ADDISS was 15%, excluding those who were ineligible or responded after study completion. Excluding those who responded after the study finished, the response rate from the NHS outpatient clinics was 19%. An overview of recruitment is shown in Figure 1 and participants’ characteristics are displayed in Table 1.

**Table 1:** Demographic details

	**Diagnosed in adulthood (n = 15) N (%)**	**Diagnosed in childhood (n = 15) N (%)**
**Gender**		
Males	6 (40)	7 (47)
Females	9 (60)	8 (53)
**Age Range (years)**		
18-25	0 (0)	13 (87)
26-35	1 (7)	1 (7)
36-45	6 (40)	0 (0)
46-55	5 (33)	1 (7)
56+	3 (20)	0 (0)
**Ethnicity**		
Caucasian	12 (80)	12 (80)
White other	1 (7)	2 (13)
Asian	1 (7)	0 (0)
Black	0 (0)	1 (7)
Other	1 (7)	0 (0)
**Work Status**		
Student	2 (13)	5 (33)
Full time	4 (27)	3 (20)
Part time	5 (33)	5 (33)
Unemployed	4 (27)	2 (13)
**Living Arrangements**		
Live alone	4 (27)	3 (20)
Live with parents	1 (7)	10 (67)
Live with spouse	6 (40)	2 (13)
Live with children	4 (27)	0 (0)
**Education Status**		
GCSE or equivalent	2 (13)	6 (40)
A-level or equivalent	1 (7)	6 (40)
Undergraduate degree	6 (40)	2 (13)
Postgraduate degree or higher	6 (40)	1 (7)

Participants reported that they were diagnosed with ADHD between the ages of 6 and 57 years (mean age at diagnosis = 26 years, SD = 16.8). In participants diagnosed in adulthood, the mean age at diagnosis was 40.93 years (SD =9.32), and 11.07 years (SD = 4.50) in participants diagnosed in childhood. As expected, there were differences between participant groups in terms of age, due to inherent age differences in the subgroups of interest.

Patients self-reported medical and treatment characteristics data is reported in Table 2.

**Table 2:** Medical and treatment characteristics

	**Diagnosed in adulthood (n = 15) N (%)**	**Diagnosed in childhood (n = 15) N (%)**
**Current Medication use**		
Using Medication	13 (87)	12 (80)
Discontinued Medication*	2 (13)	3 (20)
**Current Medication Type**		
Methylphenidate	10 (77)	11 (92)
Atomoxetine	1 (8)	1 (8)
Dexamphetamine	2 (15)	0 (0)
**Duration Since Diagnosis**		
< 1 year	1 (7)	0 (0)
1-5 years	4 (27)	2 (13)
6-10 years	7 (47)	4 (27)
11-15 years	2 (13)	8 (53)
16+ years	1 (7)	1 (7)
Duration on ADHD Medication** **(n = 24)**		
< 1 year	5 (42)	3 (25)
1-5 years	4 (33)	2 (17)
6-10 years	2 (17)	5 (42)
11-15 years	0 (0)	2 (17)
16+ years	1 (8)	0 (0)

*Discontinued for a period of 6 months or longer.

**missing data for 6 participants.

The mean total score was 13 (SD = 3.20, range 6–18) out of a total of 18, which indicates moderate symptom burden across the sample. Participants diagnosed in adulthood had numerically higher mean total scores (14, SD = 2.87, range 8–18) compared to those diagnosed in childhood (12, SD = 3.47, range 6–17). The mean number of items which indicated that ADHD impairment was definitely present was 5 out of a total of 10 (SD = 2.95, range = 0-9). Participants diagnosed in adulthood had numerically higher mean scores for impairment (6, SD = 2.55, range 1–9) than those diagnosed in childhood (4, SD = 3.14, range 0–9). Twenty seven per cent of the sample reported mild symptom severity, 63% reported moderate and 10% reported severe symptom severity. This demonstrates that the sample included a range of different symptom severities.

Five meta-themes emerged from the qualitative data: ‘*An uphill struggle’: the challenge of accessing services,****‘****Accumulated Psychosocial Burden and the Impact of ADHD****’****,****‘****Weighing up Costs vs. Benefits of ADHD Pharmacological Treatment’, ‘Value of Non-pharmacological Treatment’ and ‘Barriers to Treatment Adherence’* with a common thread of ‘*Unmet needs’* which runs throughout participants’ experiences of treatment and accessing services.

These themes and subthemes are presented below, with illustrative quotes from study participants.

### Theme 1: *‘An uphill struggle’*: the challenge of accessing services

#### Perceived barriers to accessing care

Getting a diagnosis and accessing ADHD services was often a long and arduous process, particularly in those diagnosed in adulthood (“*I had to fight tooth and nail for two years to get this on the NHS, it was extremely difficult”* P4). Many faced multiple barriers to getting care, often perceived by patients to be due to overwhelmingly negative and sceptical attitudes towards ADHD by health professionals. Some General Practitioners (GPs) were perceived by patients as unwilling to refer to specialist services because of disbelief in the condition or poor awareness of adult ADHD (*“It was an uphill struggle”* P3). Getting a definitive diagnosis of ADHD was a challenge in those without access to specialist care. In a few who initially accessed adult ADHD care in countries outside the UK (mainly in the United States); there was a stark contrast between these positive experiences and the difficulties encountered upon accessing continued care in England.

Participants reported that the struggle of getting access to services in adulthood exacerbated their feelings of disempowerment, distress and helplessness and led to a downward spiral in functioning. In several cases, delays in accessing care left participants feeling unable to cope, prompting periods of severe functional impairment, depressive mood and even suicidal ideation (“*Putting somebody with ADHD through a bureaucracy is torture.... it’s like treating a diabetic in a bakery”* P21). Due to these emotional difficulties, a few with particularly negative experiences of accessing care even considered whether getting a diagnosis was worthwhile, because of the lack of subsequent support received (“*I was very lucky to get a diagnosis, but the diagnosis really is not very helpful without appropriate support… it’s disgraceful there just isn’t a system”* P2).

Accessing prescription medication for ADHD was also problematic, as some patients reported GPs being unwilling to prescribe, or had encountered pharmacists who were reluctant to stock or dispense ADHD medication. Therefore, some had to take multiple trips to the pharmacy every month to collect medications, which was stressful due to difficulties in getting organised and remembering (“*the psychiatrists but often mostly pharmacists act as if [ADHD drug] is plutonium and it’s, like, ridiculous”* P21). Some participants recalled periods of time where their doctor refused to prescribe the medication for them; this varied from several days to several years. Patients reported that this was often due to doctors who held negative attitudes towards prescribing ADHD medication or health trusts that refused to fund medication. These periods of enforced cessation were highly distressing, and long periods without medication led to severe emotional distress and functional impairment in some.

Some reported problems with transitioning from child to adult services, such as discontinuation of treatment or support upon reaching 18, or delays with referrals to adult services (*“There are places you can go as a kid, but not as an adult, it’s kind of swept under the carpet as soon as you reach 18”* P15). Overall, more positive experiences of using ADHD services in adulthood were reported from those diagnosed during childhood, or with access to private healthcare.

#### Doctor-patient communication issues

A few participants experienced problems communicating difficulties associated with their ADHD to their GP, and reported becoming overly emotional during initial consultations (*“He’d [GP] just seen me crying and shaking and saying I have difficulty concentrating so I don’t blame him for thinking I was in pieces really”* P5). Some participants were distressed at being unable to access ADHD specialist care and being placed on repeat prescriptions by their GP, without monitoring or support. This was deemed inadequate at meeting their needs and resulted in feelings of abandonment by the healthcare system, especially in those treated since childhood. For example, many participants whose primary interaction was with a GP strongly desired support with adjusting their medication type and dosage, or advice regarding coping with side effects, but did not receive it. Evidently, those with little support by health professionals reported poorer self-efficacy (confidence in their ability to effectively self-manage their condition).

In those who had experienced severe difficulties accessing care, a few participants felt unwilling to disclose the negative effects of pharmacological treatment to physicians over fears of medication being withheld (*“I got quite bad [side effects], but I didn’t want to tell the GP that because any excuse, I felt any excuse they’d have to stop the meds…so that was rather miserable for a time”* P3). Some desired greater involvement in treatment decision-making and access to a wider range of ADHD medication in order to improve functioning and reduce side effects. In contrast, those with access to ADHD specialist care reported that they received and strongly valued this type of support.

Patients discussed several unmet needs regarding consultations with psychiatrists. Many wanted to receive more informed advice to help shape realistic expectations of both short and long-term medication effects, as some detected reluctance amongst healthcare professionals to discuss potential risks. A few participants felt that consultations with psychiatrists focused too strongly on the impact of drug treatments, rather than on the condition itself, which was thought inappropriate for adequately supporting those diagnosed later in life. Therefore, some felt that psychiatrists should assume a more holistic approach to care and also focus on the psychosocial impact of ADHD.

Many participants desired more frequent monitoring and support, although others did already receive this. Some participants perceived an unwillingness amongst health professionals to help patients adjust medication types or dosages, which was deemed important to find optimal treatment and reduce side effects (*“there isn’t really a kind of willingness or the time available or the knowledge available to tweak around with it [medication]”* P2). In addition, participants often experienced problems remembering to attend healthcare appointments, and they felt the need for easier access to care, such as more telephone follow-up consultations.

### Theme 2: accumulated psychosocial burden and the impact of ADHD

#### Accumulated psychosocial burden from a delayed diagnosis

Participants who went undiagnosed until adulthood often initially sought help from health care services due to depression or anxiety complaints, which resulted in years of misdiagnoses. The cause of co-occurring disorders was attributed by many of these patients to the underlying ADHD, as they reported long-term ADHD symptoms since childhood. Many reported that treatments for these disorders were ineffective as they failed to address core symptoms of the ADHD. Those diagnosed from later adolescence onwards perceived this as a ‘late’ diagnosis and felt a strong sense of regret and frustration over a lack of earlier detection (*“I just think had things been understood at a younger age, I wouldn’t constantly feel that I was a failure, I was useless, I was sort of not worthy or I was never going to achieve anything”… “because it was such a late diagnosis, the damage is essentially done”* P9).

In most participants diagnosed in adulthood, living with undiagnosed ADHD had led to an accumulated psychosocial burden due to a chronic sense of failure and missed potential in many areas of life (“*I think that’s probably the biggest thing… is the accumulation of shame and failed whatever, education, jobs, relationships, there’s sort of an accumulation”* P2). The emotional impact of living with undiagnosed ADHD had led some to psychological breakdown and suicidal ideation as they felt unable to cope with the burden of impairment. Prominent feelings of low self-worth in some participants were impacted by a sense of underachievement as well as having experienced on-going negative criticism and labelling by others.

#### The impact of ADHD: a chaotic life

The impact of ADHD impairment was not limited to those with a delayed diagnosis, but affected the whole sample. In many participants, ADHD related impairment had an overwhelmingly chaotic impact on every aspect of patients’ lives. Many had struggled with the transition into independent adult life, and often felt ill equipped to cope (*“It affects you in every single way possible”* P26). For many participants, a chronic sense of disorganisation was particularly debilitating, due to symptoms of poor concentration and memory (*“Personal organisation is catastrophic, it’s not good....I can’t organise my way out of a room.... essays, dissertations, anything I spend 20 times the amount of time that someone of my general level of brain power ought to take, I simply cannot organise stuff in my head”* P4). Home and working lives were often chaotic as many participants constantly struggled with completing routine tasks, chronic forgetfulness, prioritising or managing time appropriately.

Most participants recalled difficulties with achieving academically at school or university. The impact of ADHD culminated in a sense of underachievement in many aspects of participants’ lives, which was more evident in those diagnosed in adulthood, although some diagnosed during childhood still expressed feelings of unfulfilled potential despite an earlier diagnosis and treatment. Although several participants diagnosed in adulthood were highly educated, many reported a sense of underachievement in other areas of life such as interpersonal relationships or in work roles, for example.

ADHD had a considerable impact on work, as some struggled to find suitable work roles. Four participants who were graduates were unemployed, citing ADHD difficulties as the reason. Of those working, many felt inefficient at work due to difficulties with procrastination, perfectionism and concentration and felt that their work output was poor. The negative impact of ADHD on participants’ working lives appeared greater in those with a delayed diagnosis, although many in the childhood group were still students.

Social impairment was discussed by many, as participants reported frequent conflict and relationship breakdown, particularly those diagnosed in adulthood. Many had problems with impulsively blurting inappropriate comments and often felt misinterpreted by others as ‘blunt’ or ‘rude’. Social impairment meant that many had difficulty maintaining long-term friendships. Some discussed the deleterious impact of impulsivity in terms of previous drug and alcohol abuse, reckless decision-making and involvement in criminal behaviour, which was reported by participants as being detrimental to relationships, finances and personal safety.

Emotional impairment was evident in all participants, particularly overreactions of frustration or anger, as many had difficulty coping with emotions which could become overwhelming. Some experienced periods of emotional breakdown and functional disability, as they were unable to complete simple tasks. According to participants, ADHD was detrimental to physical wellbeing, due to difficulties transitioning from sleep to wake and vice versa. Participants diagnosed in childhood perceived certain ADHD symptoms to have lessened since adolescence, particularly hyperactivity, although many reported feelings of restlessness or being constantly *“on the go”* (P24). A few female participants reported internalised hyperactivity, such as overactive thoughts and finding it difficult to relax (*“My mind is always working and I can never rest, everything competes for my attention”* P9). Overall, the burden of impairment appeared greater in those with a delayed diagnosis (from late adolescence onwards), and positive adjustment to ADHD seemed to be facilitated by an earlier age at diagnosis.

### Theme 3: weighing up costs vs. benefits of ADHD pharmacological treatment

#### Benefits of ADHD medication: reducing the chaos

All participants discussed the benefits of pharmacological treatment, as many felt medication lessened the chaotic impact of ADHD, such as disorganisation and procrastination. Some participants felt that their cognitive abilities and memory were improved while on medication and several thought it improved their ability to focus on tasks (“*it was like putting on a pair of glasses for vision; I found them excellent when it comes to concentration…it does lift the fog”* P14). Medication also improved feelings of normalcy (*“I felt normal [on medication] and the rest of the time I felt like I was on drugs”* P5). Drug treatment was often perceived as vital to daily functioning and was deemed necessary at improving focus in work or educational situations involving routine tasks or written assignments. The impact on social impairment was varied, as some felt medication enabled them to function better in social situations. Medication was thought to be beneficial at reducing emotional impairment, particularly in younger participants who felt more able to control anger or restlessness and hyperactivity.

#### Perceived costs of ADHD medication

Participants also weighed up the negative aspects of pharmacological treatment. Around two thirds of the sample reported side effects, which varied greatly between participants, such as insomnia, headaches or poor appetite. A few reported severely impairing side effects such as paranoia and many experienced daily withdrawal symptoms as medication wore off. Some participants perceived a loss of self-identity, and felt that medication diminished positive aspects of ADHD, such as sociability (“*When I’m on my tablets I’m a totally different person. I can’t socialise. I am so socially awkward”* P11).

A few treated from an early age felt that medication effectiveness reduced in the long-term, which meant changing drug type every few years. More negative beliefs towards drug effectiveness were found in those who had received inadequate specialist support to find optimum treatment (“*I need to know the reasons why I’m taking it [medication] and what effects I can expect because otherwise the negative sides of things outweigh it”* P10). In addition, a few participants expressed disappointment over medication effects, and perceived a lack of long-term improvement, particularly in the group diagnosed in adulthood that had medication as a standalone treatment. Some participants who felt that the disadvantages of medication outweighed the advantages, were also more often in this group, than those diagnosed in childhood. A few participants also reported overenthusiastic psychiatrists raising overly high expectations of medication effectiveness.

#### Perceived limitations of medication for ADHD

Overall, although most participants felt that drug treatment was necessary at alleviating ADHD impairment (“*It’s been a life saver for me”* P21), many participants, particularly those diagnosed from late adolescence onwards, felt that medication as a standalone treatment was limited. These participants reported a plethora of unmet psychosocial needs as many strongly desired additional psychological or educational support alongside medication to improve functioning. However, few had access to non-pharmacological treatment (“*I think it’s effective [medication] for say maybe 30% of the condition. 30% of the condition it doesn’t deal with and the other 30% is actually the effect the condition has had on you all your life without knowing’* …‘*so it’s really, really good but it’s not the whole story and it’s not enough. There needs to be more work on counselling”* P19). Most participants felt that the system of care should offer more holistic treatment programs (*“I mean meds help but they’re not the entire answer....it’s the stuff other than the meds that’s there’s a real gaping hole and it’s got to tie in together…that sort of holistic management”* P3). In particular, improving organisational and coping skills, reducing stress and alleviating social and emotional impairments was deemed necessary by many.

### Theme 4: perceived value of non-pharmacological treatment

Equal value was placed on psychosocial and pharmacological treatments by those with experience of both treatment types, who felt that medication alongside additional psychosocial support was particularly effective (“*So beyond medication I am not getting any interactive care [CBT] which I have always found is as important as the medication”* P28). Treatments such as Cognitive Behavioural Therapy (CBT), Counselling or Life Coaching were deemed beneficial by some, particularly at helping patients learn practical coping strategies and deal with the psychosocial burden. The social element of group therapy or support sessions was highly valued, enabling participants to learn how others coped with ADHD (*“The medication has been a great help but I know when I went through the group CBT and when you got speaking to so many different people it was great because you also got to learn how they cope and how they can deal with it”* P24). Yet, some thought psychosocial treatments should be better designed and more appropriate for ADHD patients in terms of being more engaging and accessible. Participants diagnosed in childhood reported greater access to psychosocial treatments whilst in child services, but these were often discontinued in adulthood. However, despite reporting greater access, these participants placed less value on the usefulness of non-pharmacological treatments, compared to participants diagnosed in adulthood. A few felt the timing of psychosocial treatments was inappropriate in adolescence and would be more useful in helping them to cope with adulthood challenges (*“I could go back and say oh I need help with this now, so whether it was too early, the CBT I don’t know, being at that age”* P17).

### Theme 5: barriers to treatment adherence

Issues of medication non-adherence (e.g. missing doses) and experimenting with treatment cessation (longer periods of medication cessation) also arose in the interviews. Participants gave several explanations for occasionally not taking their medication as prescribed. The most commonly reported reason for non-adherence was forgetfulness but other reasons included only taking the medication when it was perceived as needed (*“I just take them for exams now or if there’s something going on that I find a struggle”* P1), not liking to take medication during weekends or holidays and perceived lack of guidance from clinicians *(“Because, I don’t know, and I’m not getting any guidance, I’m not using the tablets properly”* P10).

Furthermore, many participants talked about experimenting with longer periods of medication cessation. Reasons for cessation were: the experience of side effects, a desire to cope without medication, uncertainty over medication effectiveness and a sense of lost self-identity. A few participants had not been taking medication for 6 months or longer but the majority of cessation attempts resulted in restarting treatment. Participants talked about the negative impact that stopping treatment had on life at home, work, school and emotional impairment, such as increased feelings of anger, frustration and behavioural problems. In many cases, participants did not discuss cessation attempts with their physician, so subsequently received no medical support during these problematic times.

## Discussion

These findings illustrate that accessing ADHD diagnostic and treatment services is often an arduous and challenging process for adults in England. Poor awareness and inappropriately high levels of scepticism among health professionals was encountered by participants, despite recent national clinical guidelines and consensus among experts about the diagnosis and treatment of ADHD in adults [2,21]. On-going difficulties with accessing ADHD medication was evident, as many perceived fearful attitudes and reluctance amongst pharmacists and GPs towards prescribing; this is in line with findings from a recent review [31]. The lack of licensed ADHD medications approved for use in adults during the time of this research may have contributed to this reluctance to prescribe, although NICE has recommended the use of unlicensed ADHD medications since 2008 [21]. Severe functional and psychological impairments were reported by participants who had found that accessing care was particularly challenging. As previous research demonstrates that maladaptive coping strategies in dealing with stressful situations are present in adults with ADHD compared to people without ADHD [17], this indicates that adults with ADHD may be particularly vulnerable to system-related impairment, which can have a deleterious impact.

Importantly, this study compared adult patients diagnosed with ADHD at different stages of the lifespan, and the psychosocial burden of ADHD related impairments appeared more severe and problematic in those diagnosed from later adolescence onwards (16 years or older), who deemed this as a ‘late’ diagnosis. Based on participants overall accounts of managing their ADHD, those diagnosed in childhood appeared more positively adjusted to the condition and reported fewer psychosocial and service-related unmet needs, compared to those diagnosed in adulthood. However some of those diagnosed in childhood did experience problems transitioning from child to adult services; similar to findings reported previously which suggests that pharmacological treatment in young adults with ADHD may be discontinued prematurely by health care professionals [32,33]. Evidently, ADHD related impairment had an overwhelmingly chaotic impact on all aspects of participants’ lives, both those diagnosed in childhood and adulthood, supported by previous research [1,10-12,34]. Despite the unusually high levels of educational achievement in those diagnosed in adulthood (80% had a Bachelor degree or higher), many still had an overriding sense of unfulfilled potential across multiple areas of life, such as interpersonal relationships and work achievements. Although the group diagnosed in childhood had much lower educational achievements in comparison, this is most likely a reflection of the age differences between the groups as those diagnosed during adulthood were older.

This study highlights that adult patients with a delayed diagnosis of ADHD have many unmet needs regarding treatment. According to NICE guidelines [20,21], all ADHD patients should have a comprehensive treatment program, with pharmacological treatment offered (methylphenidate as first line treatment), unless patients prefer psychological treatment alone. The guidelines state that treatment should meet the psychological, behavioural and educational/or occupational needs of patients [20]. Yet in this study, many participants had no access to non-pharmacological treatment on the NHS and displayed many unmet psychosocial needs. Whilst patients felt ADHD medication was necessary to alleviate impairment, those diagnosed from late adolescence onwards expressed strong views about wanting additional psychological or educational support, as medication by itself was deemed inadequate and many struggled to cope. This was also reflected by quantitative data collected which demonstrated that symptoms and impairment were still present despite medication use. This suggests that there may be a wide gap between practice and NICE guideline policy in England, in terms of the services and support that adults with ADHD should receive [20,21].

Evidently, those patients with experience of both treatment types placed equal value on pharmacological and non-pharmacological treatments, and appeared more positively adjusted to the condition. The potential value of psycho-education, particularly when combined with medication has been highlighted in a recent review of care for children with ADHD [31]. There are relatively few studies, in comparison with pharmacological studies, that have investigated the efficacy of non-pharmacological treatments for ADHD in adult patients, but these provide promising results and indicate that this is an important area for future development [35-41].

**Figure 2:**
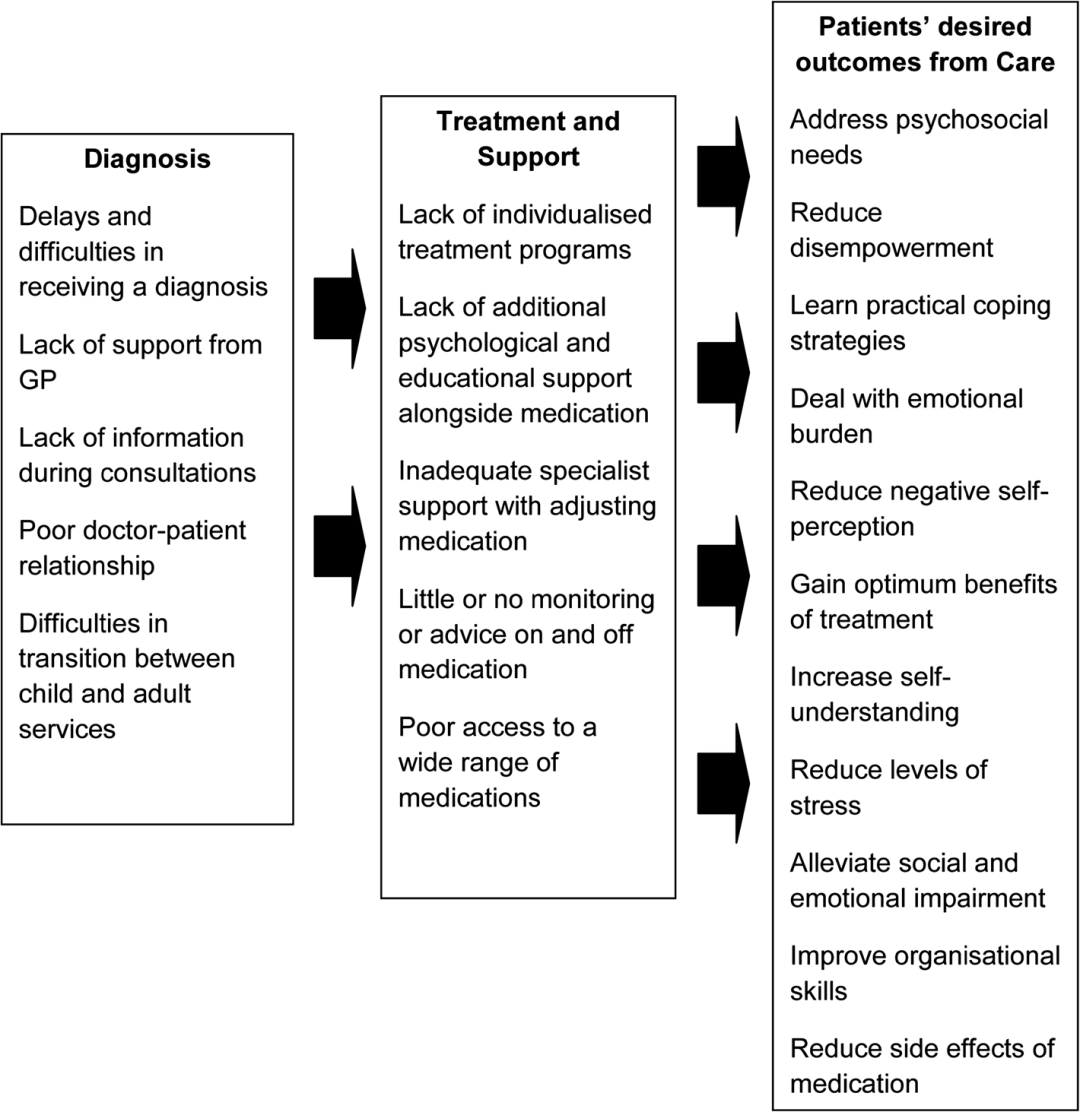
Patients’ self-reported problems regarding the ADHD system of care in England.

There were stark differences in the experiences of those with and without access to specialist ADHD care. Some participants were placed on repeat prescriptions by their GP and went unmonitored with drug regimes, which was deemed completely inadequate by participants, and is neither in line with current ADHD policy nor with NICE guidelines [20,21]. This was one of several factors in this study which contributed to poorer adherence and a sense of abandonment from services. Without a specialist to give advice on adjusting medications, participants felt the optimal benefits were not achieved and struggled to cope with side effects. Conversely, those who had received specialist help with finding their optimal dose strongly valued this support, which in most cases would also have included advice on coping strategies and psychological support. Improvements to care provision were also discussed by those with access to specialist care, and some felt that more informative advice regarding medication and greater involvement in decision-making during consultations would be beneficial so they would feel more informed about their treatment. A more holistic approach was desired by participants who were diagnosed later in adulthood, as they felt that psychosocial issues should be discussed during consultations in addition to drug treatment issues. A summary of patients’ self-reported problems and areas of unmet need related to the system of care in England is displayed in Figure 2.

There are several limitations of the current study. The findings are based on a relatively small sample size. However, this number is typical in qualitative research and thematic saturation was achieved [24]. There was also a low response rate, possibly due to the difficulties reported by the participants regarding organisational impairment as well as the possibility of outdated database records from the ADHD charity (ADDISS) that were used to identify potential participants. Difficulty in recruitment was also reported by a previous qualitative study in young adults with ADHD [42]. Furthermore, exact response rates were difficult to gauge due to uncertainty over how many potentially eligible participants read the ADDISS newsletter. Recall bias may also have occurred, particularly in those diagnosed in childhood, as participants were sometimes discussing events that happened several years ago, e.g. experience of diagnosis. Additionally, although the gender ratio of patients with ADHD is almost 1:1 in adults [43,44], there were more female participants in the sample.

Further quantitative research is warranted to assess the true extent of unmet needs in the wider adult ADHD population in the UK as well as other countries with different healthcare system. Additionally, further research is necessary to design and evaluate more appropriate psychosocial interventions for providing educational and psychological support for adults with ADHD, as there is limited research in this area. Furthermore, exploring physicians’ perceptions and experiences of treating adult ADHD patients may also indicate areas for improvement in the system of care.

The current study indicates where improvements to practice and policy are urgently needed so that patients’ needs are met. Firstly, NICE guidelines [21] need to be implemented in practice, in England, to minimise condition and system-related impairment in adults with ADHD. Health professionals may benefit from a greater awareness of adult ADHD to enable earlier detection and diagnosis when appropriate. Additionally, greater availability of psychological or educational interventions, particularly for patients with a delayed diagnosis, may help patients cope better with their ADHD. Patients with ADHD may also require additional support around early adulthood, in those with continuing impairment, to ensure patients adjust better with the transition from child to adult care. Furthermore, better continuity of care and frequent monitoring may be warranted so that adults with ADHD are better supported to achieve optimal functioning.

## Conclusions

This study highlights the challenging process of accessing adequate ADHD diagnostic and treatment services, which can have a damaging impact on adult patients with ADHD. The findings indicate that a substantial psychosocial burden exists in adult ADHD patients, particularly those diagnosed from late adolescence onwards. This study also demonstrates the potential need for better psychological and educational support for adults with ADHD, especially patients who have a delayed diagnosis. Overall, these findings highlight the wide gap between recent NICE guidelines [20,21] and current practice in England regarding the clinical care for adults with ADHD, also supported by a recent article which argued that adults with ADHD are “ignored and under-treated” by services [45].

## Abbreviations

ADHD: Attention deficit hyperactivity disorder; GP: General practitioner; NHS: National Health Service; NICE: National Institute of Health and Clinical Excellence; ADDISS: National Attention Deficit Disorder Information and Support Service; ASRS: Adult ADHD self report scale; SD: Standard deviation.

## Competing interests

LM, SC, PA and IW had support from Shire Pharmaceuticals LLC for the submitted work; PH and JS are employees of Shire Pharmaceuticals Inc, and RS was an employee of Shire Pharmaceuticals at the time the submitted work was commissioned and is currently an employee of Bayer Pharmaceuticals. PH and JS own stocks in Shire Pharmaceuticals. In the previous 3 years, LM and SC have had no other relationships or activities that could appear to have influenced the submitted work. PA has conducted consultancy work for Shire Pharmaceuticals, Eli Lilly, Janssen Cilag and Flynn Pharma. PA has grants/grants pending with Shire Pharmaceuticals, Janssen Cilag, Vifor and QB Tech. PA has received payment for lectures from Shire Pharmaceuticals, Flynn Pharma, Janssen Cilag, Eli Lilly and Medice. In the previous 3 years, IW has conducted consultancy work for Shire Pharmaceuticals, and received payment for lectures by Janssen Cilag.

## Authors’ contributions

SC, IW and PA initiated the idea for the study and led development of the protocol, securing of funding and ethical approval. RS, PH and JS contributed to the protocol. LM and SC were responsible for the study administration, recruitment, data collection, data analysis, interpretation of results, and writing of the paper. LM and SC were the only authors who had full access to all the data in the study and can take responsibility for the integrity of the data and the accuracy of the data analysis. LM and SC wrote the initial draft of the paper, to which all the authors contributed. All authors read and approved the final manuscript.

## Authors’ information

LM has a background in Health Psychology and was a Research Assistant at The School of Pharmacy at the time the study was conducted and the paper written, and is now a PhD student at Oxford Brookes University; PA is a Professor of Molecular Psychiatry; IW is a Professor of Pharmacy; PH and JS are employees of Shire Pharmaceuticals with backgrounds in pharmacy; RS was an employee of Shire Pharmaceuticals at the time the study was conducted and the paper written, and is now an employee of Bayer Pharmaceuticals; SC was a Lecturer in Medicines in Health at The School of Pharmacy at the time this study was conducted and the paper written, and is now a Research Scientist in Outcomes Research at United BioSource Corporation.

## Pre-publication history

The pre-publication history for this paper can be accessed here:

http://www.biomedcentral.com/1472-6963/13/184/prepub
